# Microscale Liquid Transport in Polycrystalline Inverse Opals across Grain Boundaries

**DOI:** 10.1038/s41598-017-10791-3

**Published:** 2017-09-05

**Authors:** Q. N. Pham, M. T. Barako, J. Tice, Y. Won

**Affiliations:** 1 Department of Mechanical and Aerospace Engineering, University of California-Irvine, Irvine, CA 92697 USA; 2 NG Next, Northrop Grumman Aerospace Systems, Redondo Beach, CA 90278 USA

## Abstract

Delivering liquid through the void spaces in porous metals is a daunting challenge for a variety of emerging interface technologies ranging from battery electrodes to evaporation surfaces. Hydraulic transport characteristics of well-ordered porous media are governed by the pore distribution, porosity, and morphology. Much like energy transport in polycrystalline solids, hydraulic transport in semi-ordered porous media is predominantly limited by defects and grain boundaries. Here, we report the wicking performances for porous copper inverse opals having pore diameters from 300 to 1000 nm by measuring the capillary-driven liquid rise. The capillary performance parameter within single crystal domain (K_*ij*_/R_*eff*_ = 10^−3^ to 10^−2^ µm) is an order of magnitude greater than the collective polycrystal (K_*eff*_/R_*eff*_ = ~10^−5^ to 10^−3^ µm) due to the hydraulic resistances (i.e. grain boundaries between individual grains). Inspired by the heterogeneity found in biological systems, we report that the capillary performance parameter of gradient porous copper (K_*eff*_/R_*eff*_ = ~10^−3^ µm), comparable to that of single crystals, overcomes hydraulic resistances through providing additional hydraulic routes in three dimensions. The understanding of microscopic liquid transport physics through porous crystals and across grain boundaries will help to pave the way for the spatial design of next-generation heterogeneous porous media.

## Introduction

Porous materials are used as active surfaces that require energy transport through a solid and energy exchange across a large solid-fluid interfacial surface area. The rational design of porous media is often employed to create unique combinations of thermal, electrical, and fluidic transport for performance breakthroughs in applications ranging from electrode-electrolyte interfaces in batteries^1, 2^ to capillary-fed heat pipes and vapor chambers^3–5^. The microstructure of capillary-fed porous media has traditionally been amorphous, meaning that the pore distribution is irregular with no well-defined unit cell^6–12^. Due to the lack of crystalline order in these porous media, it is more challenging to accurately predict fluidic transport and precisely tune structural characteristics. These media also present enormous hydraulic resistance due to the highly tortuous networks between pores. Other porous materials used for capillary-fed wicking such as silicon pillars possess highly ordered microscale features^13–15^. However, such traditional microfabricated structures exhibit limitation in structural material choices and modulation of three-dimensional features. More recent approaches leverage template-assisted self-assembly nanofabrication^16–18^ to create novel architectures of crystalline and semi-crystalline porous media. Nanofabrication through templating provides tunability of the pore morphology (*i.e*. porosity, distribution of pore sizes, and structural arrangement) and is often an inherently scalable technique. Despite the self-assembly of well-ordered templates at small length scale (10 *µ*m), such materials are often semi-ordered due to uncontrollable defects that form between crystalline domains at larger length scale, creating porous media with polycrystalline characteristics^19^.

**Figure 1:**
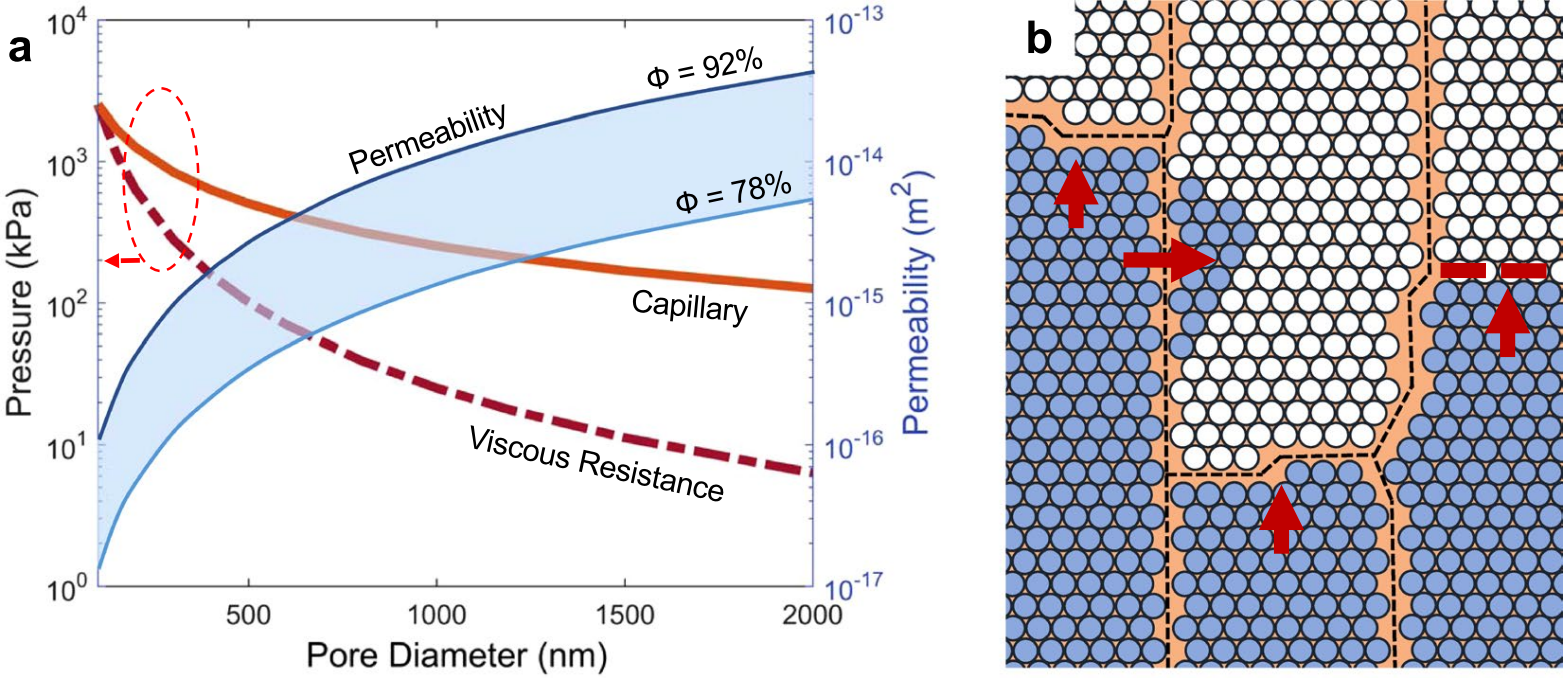
Fluid transport physics in single crystals and polycrystalline inverse opals governed by pore morphologies and grain boundaries. (**a**) Fluid transport parameters of capillary forces (solid orange line), viscous resistance (dashed red line), and permeability range (filled blue band) for varying pore diameter *D*_*pore*_ and porosity *ϕ*. The red dashed circle indicates that the capillary and viscous resistance values can be interpreted with the pressure drop on the left y-axis. (**b**) Illustration of wall-like “grain boundaries” between crystalline gains. Such grain boundaries inhibit fluid flow by causing hydraulic resistance in the pathway, resulting in lower capillary performance of polycrystals. The investigation of each individual crystalline grain enables the estimation of capillary performance of single crystals.

Inverse opals (IOs)^20–27^ are crystalline porous medium consisting of uniform pore size arranged in periodic order and have been used in a wide range of applications, including photonic crystals^21–23^, battery electrodes^24, 25^, sensors^28, 29^, and thermofluidic systems such as microfluidic heat exchanger^11, 12, 26, 27^. During self-assembly of sacrificial spheres, the “necks” between adjacent spheres in the template become the interconnected windows between the pores, the “via”, to create a continuous, fluid-permeable network of pores. Due to the combination of periodicity, interconnectivity, and sub-micron pore sizes^20^, IOs possess both high permeability and large surface area-per-volume that are ideal for mass and heat transfer. The fluidic transport through regular unit cells consisting of pores in a close packing arrangement can be easily predicted using finite element models with symmetric boundary conditions^26, 30^ (Fig. 1a). However, uncontrollable defects within such periodic array of pores inhibit normal mass flow to cause deviation from predicted transport phenomena. The inversion of the cracks within the opal template becomes an impermeable solid feature in the IO after the electrodeposition^31^ (see Supplementary Fig. S1), causing significant hydraulic resistance in fluid transport as illustrated in Fig. 1b.

In this study, we investigate the capillary-driven flow in both single crystal domains and polycrystalline copper IOs that have well-defined structure-property relations due to the hexagonal-packed pores. The capillaries of single crystal domains are compared to those of polycrystalline transport, which are governed by individual domains separated by distinct “grain boundaries.” This work establishes fundamental insights toward understanding, predicting, and engineering hydraulic transport through monoporous (*i.e*. uniform pore size and spatial distribution) crystalline IOs by revealing the effect of hydraulic resistance associated with crystalline defects on microfluidic transport. Furthermore, we extend the understanding of liquid delivery through heterogeneous (*i.e*. spatially-varying pore size) porous media^32, 33^ in the presence of crystalline defects.

### Crystalline Copper Inverse Opals

**Figure 2:**
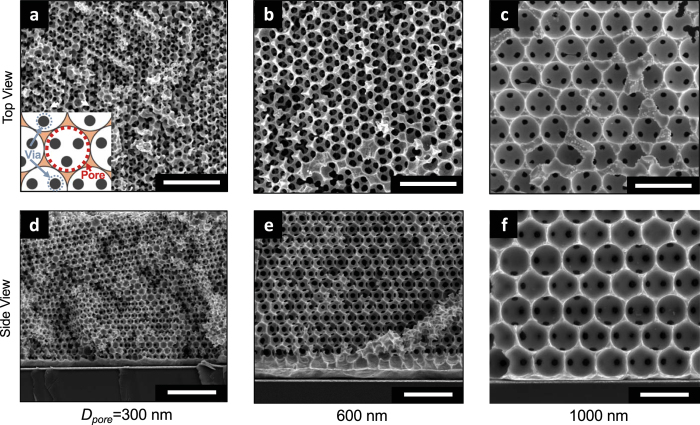
Representative scanning electronic microscope images of crystalline copper inverse opals. The periodicity of the crystalline pores with pore diameters of 300, 600, and 1000 nm is shown from the (**a**–**c**) top and (**d**,**e**) cross-sectional view. The schematic inset in (**a**) illustrates the via within each pore as the interconnected window between adjacent pores, allowing fluid to permeate throughout the porous matrix. All scale bars are 2 µm.

Crystalline copper IOs are fabricated by employing a templated electrodeposition technique (see Methods). In brief, sacrificial polystyrene spheres are self-assembled using vertical deposition, and electrodeposited copper fills the void volume between the spheres. Removal of the templated spheres reveals a copper IO containing a periodic arrangement of pores. The pore diameters of copper IOs are selected from 300 nm to 1000 nm to investigate the pore size effects on capillary wicking (as seen in Fig. 1a). Representative scanning electron microscope (SEM) images of the various pore diameters are shown in Fig. 2.

During vertical deposition of self-assembled spheres, cracks form as the opal crystal dries and shrinks^34, 35^. While opals possess well-ordered pore arrangement at small length scale, formation of defects disrupt pore packing at larger scale^19, 36^. When electrodeposition occurs, cracks in the template result in solid copper barriers that separate crystalline grain domains with an impermeable wall-like structure, defined as a grain boundary in this study. Previous simulation models and studies that are used to predict mass transport^37^ through crystalline porous media often assume no structural defects. Here, we report the capillary performance of both within single crystals and polycrystals as well as the errors of predictive modeling due to crystalline defects.

### Capillary Performance of Monoporous Copper Inverse Opals

In wicking dynamics, the capillary force scales inversely with pore diameter while viscous resistance scales inversely with the square of pore diameter *D*_*pore*_^37^. The capillary force is defined as hydrostatic-driven pressure in transporting liquid and can be calculated using the Young-Laplace capillary equation 4σcos*θ*/*D*_*pore*_, where σ is the surface tension and *θ* is the static contact angle that measures the wetting tension on the surface. Based on Darcy’s Law, the viscous pressure is a function of wicking material parameters, and the flow rate is related to the evaporation mass flux of the system. Assuming constant wick design parameters, the viscous resistance is plotted in relation to the capillary force in Fig. 1a. The balance between capillary force and viscous resistance results in a net capillary performance known as the permeability *K*. The permeability values of the porous medium are also separately computed using computational fluid dynamics (CFD) simulation models (see Supplementary Fig. S2 for calculation details). This computation gives us the correlation of porosity *ϕ* to permeability *K* = *D*_*pore*_^2^ (0.07*ϕ* ^2^−0.0539*ϕ*) for 78% < *ϕ* < *92%*. In order to account for the nonuniform annealing effects of self-assembled spheres, the maximum and minimum *ϕ* are used to determine the upper and lower limit of permeability, respectively, as indicated in the blue band in Fig. 1a. The computed range of permeability demonstrates the interdependent wicking mechanisms of permeability, capillary, and viscous resistance pressure on pore morphologies such as porosity and pore sizes.

**Figure 3:**
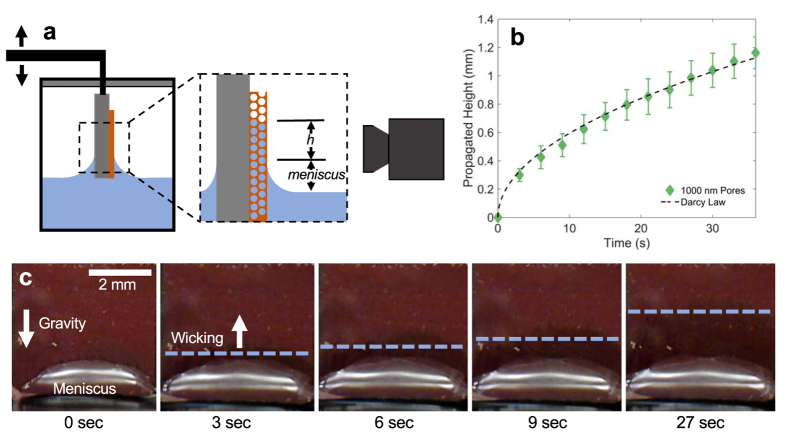
Visualization of capillary wicking tests. (**a**) A schematic for the experimental set-up of the capillary wicking. The propagated liquid rises as a function of time which is captured with a camera and then plotted. (**b**) A representative plot of a typical measured wicking height *h* from a copper IO with a pore diameter of 1000 nm. The dashed line is the numerical trend estimated using Darcy’s Law. (**c**) Time evolution images of a copper IO wicking up deionized water. The blue line is a guide for the effective wicking height *h*_*eff*_ starting from the average of the meniscus’s top edge.

**Figure 4:**
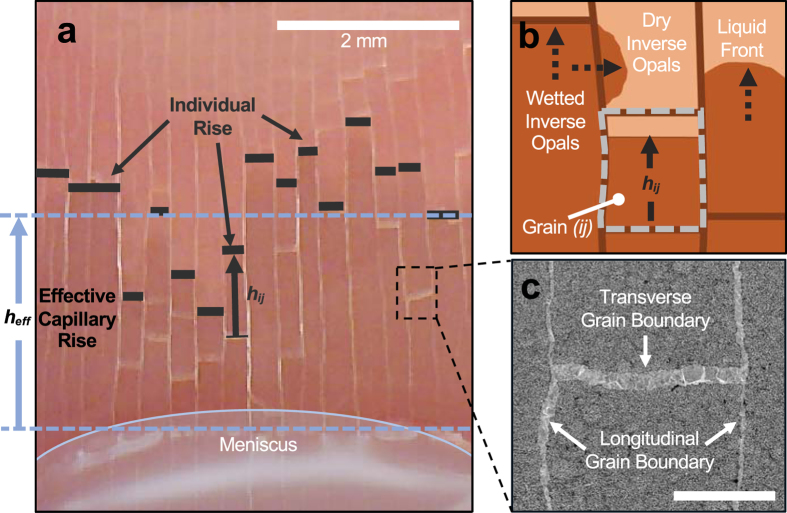
Visualization and illustration of microfluidic transport in polycrystalline inverse opals showing individual grain domains and grain boundaries. (**a**) A still photo of a vertically placed copper IO wicking up liquid from a reservoir. Due to grain boundary defects, propagated height through each grain column varies across the width of the sample. The average of the general propagated height is determined as the effective capillary rise *h*_*eff*_. See Supplementary Movie for the details. (**b**) The illustration of individual grain domains and grain boundaries. The capillary rise *h*_*ij*_ within individual grain *ij* can also be obtained as a function of time *t*_*ij*_. The observation suggests that the wicked liquid can transverse around the micro-obstruction defects. (**c**) Microscopic image showing individual crystalline domains separated by a set of transverse and longitudinal grain boundaries. The scale bar is 100 µm.

**Figure 5:**
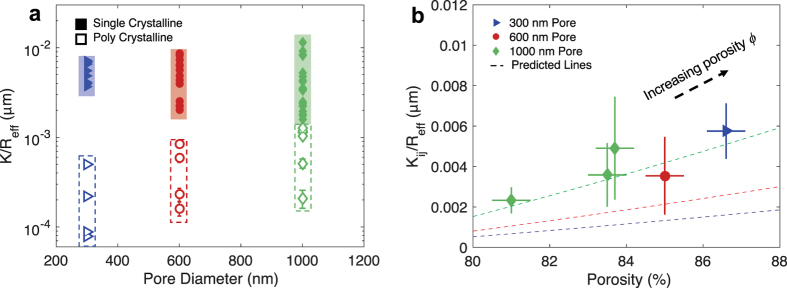
The key figure of merit, the capillary performance parameter (defined as *K/R*_*eff*_), for both single and polycrystalline. (**a**) The capillary performance parameter of the individual crystalline grains of 10^−3^ to 10^−2^ µm (filled markers) is superior to that of polycrystalline samples ranging 10^−5^ to 10^−3^ µm (unfilled markers). (**b**) The capillary performance parameter of single crystalline grains as a function of porosity *ϕ*. The dashed lines are predictions from the CFD calculations.

To evaluate the effect of pore diameter on the wicking capability of the porous structures, monoporous copper IOs are first examined using rate-of-rise of a propagating liquid front (Fig. 3 and Supplementary Movie). The key figure of merit in determining the wicking capability is based on the capillary performance parameter, which is defined as the ratio of permeability to effective capillary pore radius *K/R*_*eff*_. The capillary performance *K/R*_*eff*_ can be calculated from the capillary rise height *h* and the corresponding time *t* using Washburn dynamics^38^. When considering the balance of capillary driving forces and viscous resistance while neglecting the effect of gravity, the capillary rise height *h* is expressed as:

1 $${h}^{2}=\frac{2\sigma }{\varphi \mu }\frac{K}{{R}_{eff}}t$$

where *µ* is viscosity of the liquid, and *K* is the permeability. The effective pore radius *R*_*eff*_ = 0.5*D*_*pore*_cos^−1^ *θ* where *θ* is the static contact angle. In order to estimate the wicking capability of the polycrystalline sample, the liquid propagated height is averaged over the width of the sample yielding the effective capillary rise *h*_*eff*_ as indicated in Fig. 4a. The calculated effective capillary performance parameter for polycrystalline *K*_*eff*_ */R*_*eff*_ ranges from ~10^−5^ to 10^−3^ µm where *K*_*eff*_ is the effective permeability (Fig. 5a). The calculated values of polycrystalline IOs are much lower than its theoretical value for single crystal as computed using our CFD models and finite element method (FEM)^12^. This might be attributed to the effects of “grain boundary defects”. The microscale wall-like structures at the grain boundary cause additional hydraulic resistance in the permeable pathway, sometimes halting liquid rise but is often observed to eventually continue across the boundaries with assistance from neighboring wetted domains (Fig. 4b).

The transport physics of crystalline porous material in the absence of grain boundaries establishes a physical upper bound of transport for a given morphology and requires a separate analysis by examining liquid rise in individual crystalline domain. Therefore, we map each crystalline grain as *ij*, such that subscript *i* and *j* represents the rows and columns where the grain is located, respectively. Once the liquid rise is observed in the individual grain, its propagating height *h*_*ij*_ is followed until the full length of the grain has been reached with time *t*_*ij*_ (see Fig. 4a,b). The capillary performance parameter of these individual grains *K*_*ij*_ */R*_*eff*_ ranges from 10^−3^ to 10^−2^ µm, such that *K*_*ij*_ represents the single crystalline permeability. The values of *K*_*ij*_ */R*_*eff*_ for single crystalline grains are consistently larger than *K*_*eff*_ */R*_*eff*_ for polycrystalline samples as plotted in Fig. 5a. These two capillary rates have approximately an order of magnitude difference because of the hydraulic resistance from the structural defects. The values of *K*_*ij*_ */R*_*eff*_ from experimental measurements and CFD calculations show a good agreement. For instance, the *K*_*ij*_ */R*_*eff*_ of 1000 nm pore diameter ranges from 10^−3^ to 10^−2^ µm, which gives us the permeability *K*_*ij*_ of 10^−15^ to 10^−14^ m^2^ while CFD calculations compute a permeability *K* of 10^−15^ to 10^−14^ m^2^ for varying porosity (decided by the annealing conditions). With increasing pore diameters from 300 to 1000 nm, the capillary performances for both single crystalline and polycrystalline samples also increase.

In this calculation, only grains of *i* = 1 (*i.e*. domains that are in contact with the meniscus region) are calculated in order to accurately compare the *K*_*ij*_ */R*_*eff*_ values of different pore diameters. By wicking fluid directly from the reservoir, the grains that are in contact with the meniscus transport liquid in the absence of grain boundaries so that the only hydraulic resistance present is within the grain domains. As the fluid is pulled across multiple structural defects with increasing wicking height, the overall hydraulic resistance increases with the number of defects that must be overcome. Additional transverse boundaries may restrict the wicking flow of the liquid from its supply source and thus, negatively affecting the resulting capillary rates of individual grains of *i*≠1. Still, the large variance of capillary performance parameter is observed between individual grains, which may be attributed to at least two influencing factors, which include 1) porosity variation between the individual grains and 2) porosity variation near the vicinity of grain boundary defects.

The porosity *ϕ* is a critical factor in determining the overall permeability of the porous matrix as shown in Fig. 1a. The porosity of the IOs can be roughly controlled through modulation of the opal template with fabrication parameters such as annealing temperature^26, 39, 40^. The porosity values can be calculated with the quantification of the normalized via diameter *D*_*via*_ */D*_*pore*_ through post-image processing and are shown to be comparable within the range of previous IO porosity measurements^12, 19, 30^ (the statistical distributions of our porosity calculation are presented in the Supplementary Fig. S3). In our calculation method for the porosity of IOs, we assume that the sintering of spheres occurs isotropically, spheres self-assemble into close-packed crystalline arrangement, and spheres remain perfectly round. Based on our assumptions, we expect the accuracy of our correlation will deviate when porosity is significantly higher than the crystalline-packing of spheres (~74%). The capillary performance parameters of the individual crystalline grains are plotted with respect to their corresponding porosities in Fig. 5b, which displays a general trend of increasing capillary performance with porosity. The presence of porosity variations between individual single crystal grains due to the nonuniform annealing conditions explains the large variance in the capillary performance parameters in Fig. 5a. The vertical error bars in Fig. 5b represent those variations. In order to explain the nonuniformity in porosity across an individual single crystal grain, the normalized via diameter are collected at different locations. When examining the via closest to the grain boundary, it is revealed that the via diameters are often small or sometimes nonexistent, deemed as the “edge effect”. The lack of pore interconnectivity around grain boundary is caused by the disruption of the crystalline packing near a propagated crack. The drying of the opal film further tightly packs the spheres inward toward the domain center and loosely packs the spheres near a grain boundary. For instance, the via of 300 nm pore diameter samples are examined near a grain boundary as well as toward the center of the grain domain. The average via diameter increases by ~10% moving away from the grain boundaries, which results in a 1% increase in the overall porosity, indicated by the horizontal error bars in Fig. 5b. We find not only that the wall-like structures provide hydraulic resistance to fluid passing over them, but the areas around grain boundaries also present regions of lower fluid permeability, contributing to the edge effect of slowing or even halting the flow of fluid. In this process, smaller pore diameter samples have preferentially smaller grain sizes, contributing to the higher density of grain boundaries (see Supplementary Fig. S3). This observation suggests the capillary performance of smaller pore diameters is dominated by edge effects. Another observed phenomenon that may contribute to the disparity between the experimental and numerical values of porosity and permeability is the assisted capillary effect from adjacent grain domains. When examining the hydraulic transport within each individual grain domain, we observe that laterally neighboring domains would occasionally assist a wicked fluid on overcoming a transverse grain boundary to continue its transport (see Supplementary Movie). We expect the interplayed wicking behaviors from bordering domains can often affect fluid propagation, which may cause variation within our experimental measurements. The microscopic hydraulic interaction between nearby grain domains presents an investigation of interest for future study. Taken altogether, mass transport through engineered porous media is primarily inhibited by morphological defects, including both grain boundaries and low-quality regions near grain boundaries. Improvements in templated porous media for capillary applications will benefit from reducing defect density through increasing the template quality.

### Multilayered Copper Inverse Opals with Graded Pores

**Figure 6:**
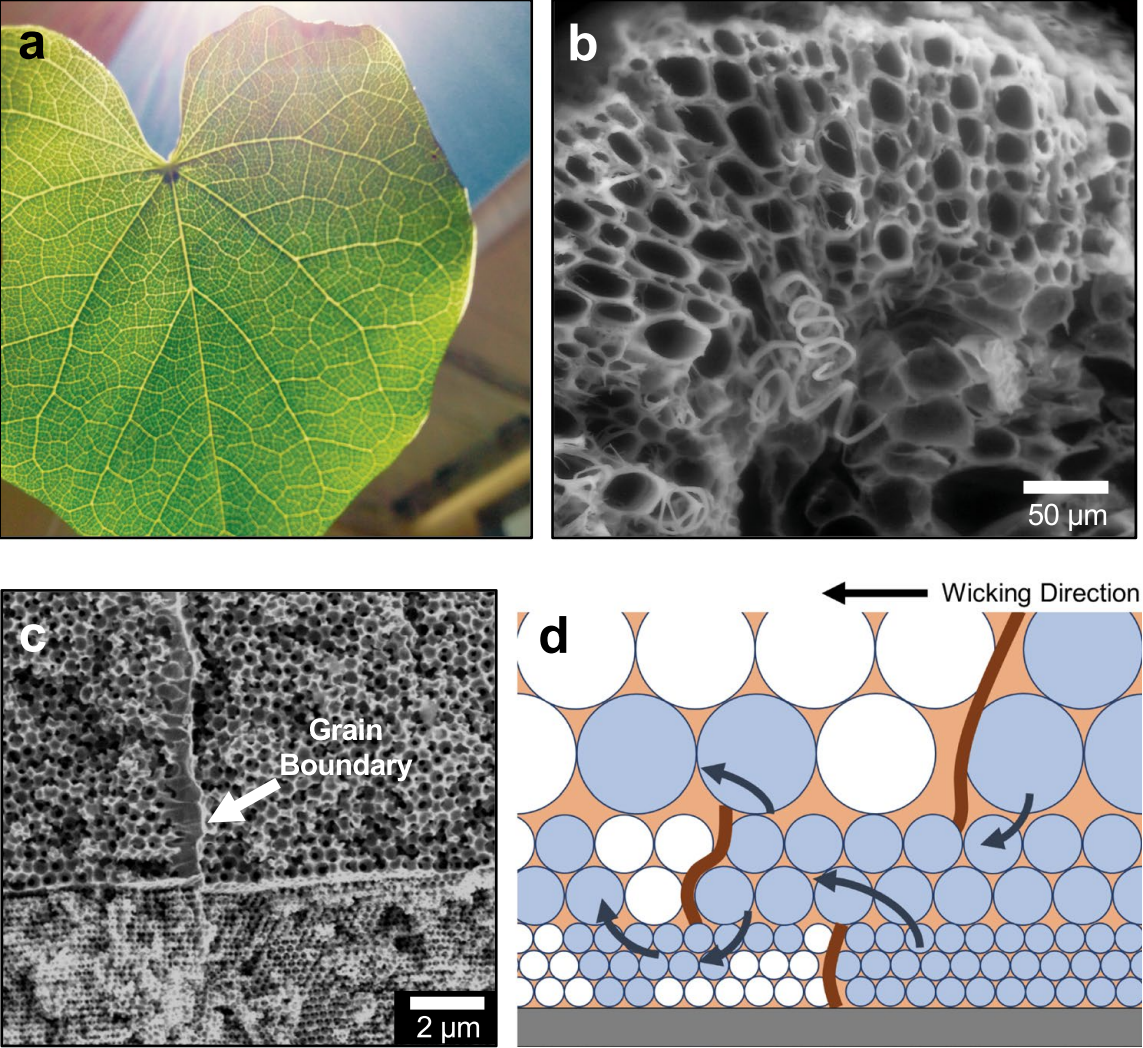
Heterogeneous porous materials in biological transport systems and gradient inverse opals. Multilength scales of porous network as seen in (**a**) plant leaf and (**b**) stem cross-section (imaged using environmental SEM) are used to emanate novel porous media with heterogeneous structural characteristics. (**c**) Biologically-inspired gradient IOs that are layered with increasing pore diameters. The grain boundaries remain within their respective crystal plane providing the three-dimensionally spatial staggering of the grain boundaries. (**d**) The illustration of spatially-varying grain boundaries shows alternative flow pathways in three dimensions, contributing to the enhancement in the effective capillary performance.

This study provides the fundamental knowledge in the wicking performance of single crystalline monoporous copper IOs, which motivates further investigation of the transport physics through three-dimensional heterogeneous porous material. Inspired by the heterogeneity in biological transport systems, such as mammalian cardiovascular networks and plant veins (see Fig. 6a,b), heterogeneous porous material is suggested to possess multiple length scale pores for optimized mass and fluid transport^41, 42^. Although several studies demonstrated the fabrication of rationally-designed heterogeneous porous crystals^32, 43, 44^, those methods utilized complicate processing and were limited by a precise control over opal films.

In this work, we successfully demonstrate gradient copper IOs by using a simple and reliable layer-by-layer growth and report the capillary performance of such gradient IO structure (Fig. 6c). Gradient multilayered opals with crystalline packing orthogonal to the substrate plane are attained through repeated convective vertical deposition to stack multiple layers with different sphere sizes. The gradient samples used in the capillary measurement show stratus composites of the individual monoporous layer of 300, 600, and 1000 nm diameter pores with each layer ~7–10 µm in thickness. The results reveal that capillary performance parameter of gradient IOs is approximately within the range of 0.6–1.1 × 10^−3^ µm (see Supplementary Fig. S4). This *K*_*eff*_ */R*_*eff*_ value is comparable to that of the best single crystal monoporous IOs (*i.e*. for 1000 nm pore diameter, a range of *K*_*ij*_ */R*_*eff*_ = 0.1–1.1 × 10^–3^ µm, see Fig. 5a) and is also superior to polycrystalline IOs by an order of magnitude. The enhanced wicking performance may be contributed to the multilayering of IOs, which provides alternative and parallel liquid pathways to circumvent a defective region as illustrated in Fig. 6d. The layer-by-layer deposition of pores also suppress these defects to mainly remain within their respective crystal plane and stagger at different regions for parallel pathways to exist.

The enhanced wicking performance of gradient copper IO demonstrates it as a nanoarchitecture with potentials to push beyond the defect-limited transport of polycrystalline porous media for applications in vapor chambers and heat pipes. Within gradient porous structures, the smaller pores provide strong capillary force that is advantageous for wicking liquid to hot spots near the substrate while the larger pores provide ventilation paths for the evaporated vapor to escape. Since cracks and defects along domain boundaries in opal films and IOs are commonly observed during the self-assembly, drying, and sintering processes, the hydraulic resistance caused by these local defects places an upper limit on the capillary performance of polycrystalline IOs. This study shows that layering multiple films of IOs can further enhance the wicking capability of these porous media by providing alternative flow pathways in three dimensions. In addition to this, the fabrication approach of copper gradient IOs presented in this work offers a new nano- and micro-architecture that provides advantageous wicking capability that can be used for developing the next-generation microfluidic and thermal management devices.

## Conclusion

In this work, we report the capillary performance parameters of both single crystalline and polycrystalline inverse opals with the aim to elucidate the effects of grain boundaries on liquid transport through porous media. Single crystalline copper IOs exhibit an order of magnitude higher capillary performance parameters, independent of the pore diameters, compared to polycrystalline copper IOs due to the dominant hydraulic resistances from the grain boundaries. Inspired by the heterogeneity in biological transport systems, we then study the capillary performance of multilayered IOs having vertically graded pore sizes. These “gradient” IOs possess capillary performance parameters that are of the same order of magnitude as single crystal grains, which may be attributed to the additional pathways in three dimensions for fluid to circumvent planar defect regions and thus providing low-resistance parallel hydraulic pathways. Further work is needed to elucidate the microscopic mechanisms of fluid flow across these grain boundaries. The fundamental physics of liquid transport in polycrystalline IOs with observable grain boundaries and defects aims to incite future predictive models for wicking capabilities of this relatively new category of rationally designed and optimized porous media.

## Methods

### Copper Inverse Opal Preparation and Surface Treatment

Monoporous and gradient copper IOs are fabricated using sacrificial template vertical deposition^45–47^ and electrodeposition method (see Supplementary Fig. S5). Titanium and gold with thickness of 20 and 80 nm, respectively, are sputtered onto silicon wafer through shadow mask with patterns of 7 mm × 5 mm rectangles, which are used as the working substrate. A self-assembled monolayer is formed on the gold surface after surface functionalization in 1 mM of aqueous sodium 3-mercapto-1-propanesulfonate for at least 24 hours. The substrates are then rinsed and immersed in suspended colloidal. The suspension is produced from monodisperse polystyrene colloids (Sigma Aldrich) diluted in deionized water with mass concentration varying from 0.04 to 0.6% w/v. By placing a hot plate underneath the well, the colloidal suspension evaporates at a controlled temperature of 55 ± 1 °C, confirmed with thermocouple at the base of the well. As the solvent evaporates, the capillary force at the surface meniscus pulls colloidal particles to the substrate for self-assembly of crystalline opals. The number of deposited opal layer is strongly governed by the changes in colloidal concentration, which is adopted in the study to tune opal thickness (see Supplementary Fig. S6).

To fabricate gradient IOs, the deposited opal film is allowed to dry and immersed in a suspension of another arbitrary sphere size, as further explained in Supplementary Fig. S5. This subsequent colloidal deposition is used to stack an additional layer of different sphere sizes and can be repeated to fabricate the desired gradient opal structure. Afterward, opal template film is annealed in a furnace oven for 5 hours at 97 ± 1 °C, monitored by a thermocouple attached to supporting copper base plate for uniform heating of the samples. The annealing process ensures adequate permeability of copper IOs since sintering the sacrificial polystyrene sphere causes them to coalesce, increasing the sphere-to-sphere contact area and thus, wider necking windows between the neighboring pores within the copper IOs. Copper is then electroplated to infiltrate the matrix of opals using potentiostatic electrodeposition (BioLogic). The thickness of the deposited copper correlates to the electrodeposition time, which can be controlled to obtain the desired number of IO layers. The templated polystyrene spheres are dissolved through immersion in a bath of tetrahydrofuran. The etchant traverses throughout the porous structure to remove polystyrene spheres through the interconnected windows between spheres. The sample is then rinsed with deionized water and confirmed with SEM imaging.

### Surface Wettability of Copper Inverse Opals

The wicking performance of the nanoscale structures is inherently influenced by the wettability of the surface. Surface wettability can be assessed by the contact angle profile of a sessile drop on solid surface. In this process, we utilize a pneumatic dispensing system to discharge droplets with volumes of ~10 nL to measure the surface energy of the copper IOs, which have pore diameters in the ranges of nanometers. A high-speed camera (100,000 fps) captures the discharge, and an imbedded software processes and tabulates the droplet contact angle on the solid surface. Approximately 10 measurements are obtained for IOs of different pore diameters. Our initial contact angle measurements of deionized water on the copper IOs reveal a hydrophobic surface (~140°). Thus, in order for porous copper medium to be viable for wicking application, the microporous copper must be further treated to lower the surface energy. In our study, the copper IOs are functionalized in 1 mM of aqueous sodium 3-mercapto-1-propanesulfonate for at least 24 hours. The surface contact angle is then remeasured, revealing a hydrophilic surface (~30–40°) ideal for microfluidic transport and assessing capillary wicking performance. The measured contact angle is used in equation (1) to calculate the *R*_*eff*_ in this study.

### Capillary Rise Measurement

The capillary wicking is measured by slowly lowering an IO sample into a reservoir of DI water using a motorized z-stage. The reservoir remains vapor saturated by enclosing the top container with parafilm that has a thin slit to let the sample through, in order to minimize evaporation from the inverse opal samples. Once the IOs are close enough to the liquid reservoir surface, a contact meniscus rapidly latches onto the substrate through wetting, during which further lowering of the *z-*stage is stopped and the time for the wicking experiment starts (*i.e. t* = 0 s). A camera with 120 fps captures the rate of propagation of the liquid front through the porous medium (refer to Fig. 3 for experimental set-up). Image frames of interest are analyzed using MATLAB software to convert the still captures into black-and-white images with appropriate thresholds. ImageJ software is then used to measure the propagated height from the top of the meniscus to the edge of the liquid front, conducting approximately 40 measurements across the sample. A width of the IO sample is predefined by the gold pattern on the substrate while SEM confirms that the thickness of the IOs remain relatively consistent for accurate wicking comparison.

### Data Availability

All data generated or analyzed during this study are included in this published article (and its Supplementary Information files).

## Electronic supplementary material

Supplementary Information

Supplemental Video
