# Supplementary material for: Microscale Liquid Transport in Polycrystalline Inverse Opals across Grain Boundaries
Source: Sci Rep. 2017 Sep 5;7:10465. doi: 10.1038/s41598-017-10791-3 (PMC5585244; doi:10.1038/s41598-017-10791-3)
Supplement: Supplementary file 1 — Supplementary Information [file 41598_2017_10791_MOESM1_ESM.pdf]

Supplementary Information

Microscale Liquid Transport in Polycrystalline Inverse Opals across Grain Boundaries

Q. N. Pham[1], M. T. Barako[1,2], J. Tice[2], Y. Won[1*]

[1]Department of Mechanical and Aerospace Engineering, University of California-Irvine, Irvine, CA 92697, USA
[2]NG Next, Northrop Grumman Aerospace Systems, Redondo Beach, CA 90278, USA
*Correspondence and requests for materials should be addressed to Y.W. (email: won@uci.edu)

1. Structural Defects in Copper Inverse Opals

While the copper IOs possess overall well-ordered arrangement, we have also observed minor defects in the inverse opal film. After the opal self-assembles, the thin film dries and shrinks which induces cracks along unstable grains. These cracks, as seen in Fig. S1a-c, are then filled with copper during electrodeposition resulting in a wall-like structure at grain boundaries (Fig. S1d-f). Such barriers cause additional hydraulic resistance in the permeable pathway.

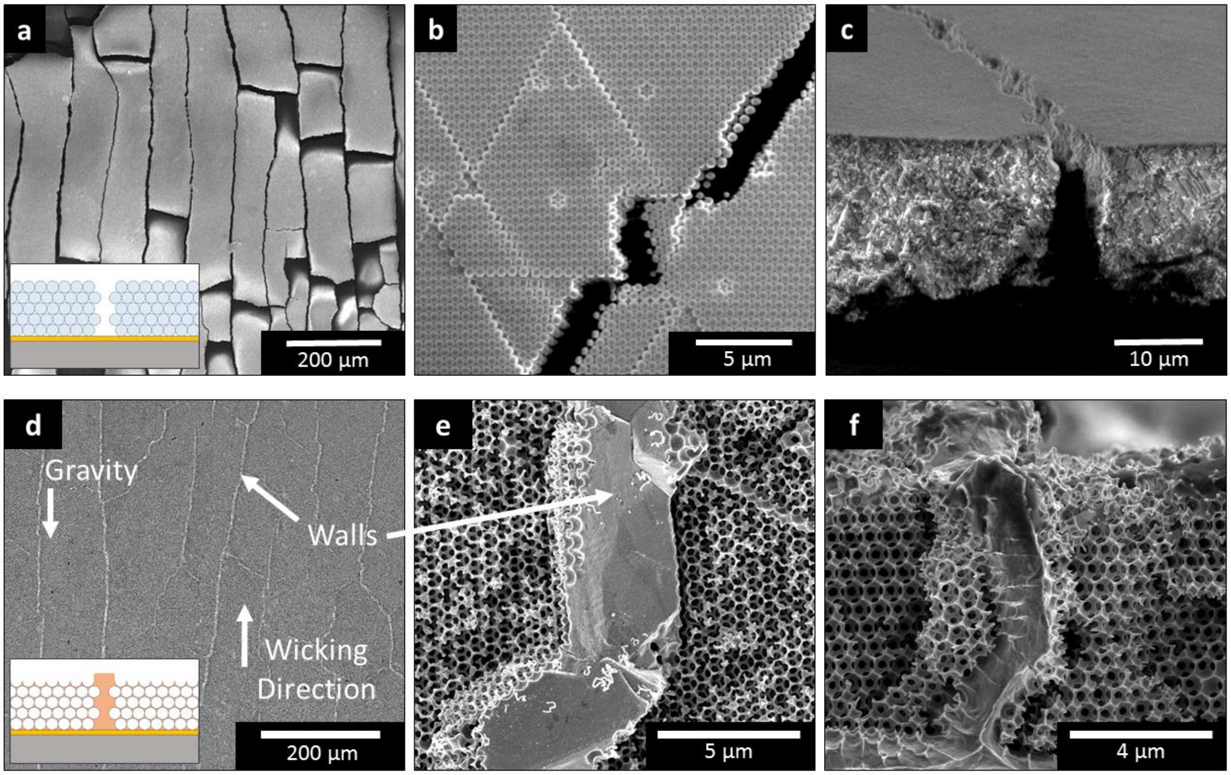

Figure S1. Characterization of cracks and subsequent walls between crystalline domains. (a-b) Top SEM images of the cracks between crystalline grains. **(c)** Side-angled SEM view of the empty space that separates two opal domains. **(d-e)** Top view of the IO after electrodeposition, where wall-like structures replace the cracks to create impermeable features along the thickness of the IO, as confirmed by the **(f)** SEM cross-section. The labels of gravity and wicking direction help to indicate the orientation of the IO sample when used for capillary wicking measurements. The insets in **(a)** and **(d)** illustrate the void crack between opal domains and the formation of an impermeable wall between IO domains after copper electrodeposition, respectively.

2. Permeability Calculation of a Unit Cell for Varying Geometry

We develop computational fluid dynamics (CFD) simulation models using ANSYS Fluent (Version 17.1) to predict the permeability of a unit cell with varying pore and via diameter. Copper IO consists of a continuous solid matrix with open pores connected to each other, forming a porous network for the fluid to pass through the medium. In such porous structures, the permeability can be calculated by the Darcy's Law[1]:

$$Q = \frac{(KA)}{\mu}\left(\frac{\Delta P}{L}\right) \qquad \text{(S1)}$$

where Q is the volumetric flow rate, A is the cross-section area of the medium, μ is the viscosity of the fluid, ΔP is the hydrostatic pressure drop, and L is the length of the sample in the flow direction. In order to calculate the permeability of copper IOs, we develop a representative unit cell of inverted face centered cubic (FCC) using ANSYS Fluent (Fig. S2a) and assuming the overlapped length between pores to range from 2% to 10% of the pore diameter (see inset of Fig. S2b). This geometry will result in the diameter of interconnecting pores from $D_{via} = 0.2D_{pore}$ with 78% porosity to from $D_{via} = 0.4D_{pore}$ with 92% porosity.

Then, a CFD simulation model is developed using a single-phase pressure-based solver assuming a steady and laminar flow as illustrated in Fig. S2a. Here, a constant velocity perpendicular to the inlet of the unit cell is introduced (to the left face) with a pressure outlet condition (at the right face). The other faces of the unit cell are set to show symmetric boundary conditions to represent the periodic nature of the unit cell within the medium. Once the pressure gradient through the unit cell is computed using SIMPLE scheme for pressure-velocity coupling, it is plugged into the Darcy's Law equation (equation (S1)) to calculate the permeability of the unit cell. In this calculation, a high mesh number is selected to minimize the mesh size effects. For instance, the mesh number used in calculating for a unit cell of D_{pore}=1.2 μm is 393,320. The computed permeability for various pore diameters ranging from 300 nm to 2000 nm is plotted in Fig. S2b.

In the classical permeability–porosity relation, the semi-empirical Kozeny–Carman model has frequently been modified in order to improve the estimation of permeability for various liquid transport media[2]. While the correlation[3] of $K=CD_{pore}^2\phi^n$ accounts for both pore diameter and porosity in porous media, we modify this equation and calculate the empirical values of C and n using the CFD results, arriving at $K=D_{pore}^2$ $(0.07\phi^2-0.0539\phi)$, which is valid for $78\% < \phi < 92\%$. The measured permeability of crystalline copper IOs and the computed values show a good agreement as explained in the main text.

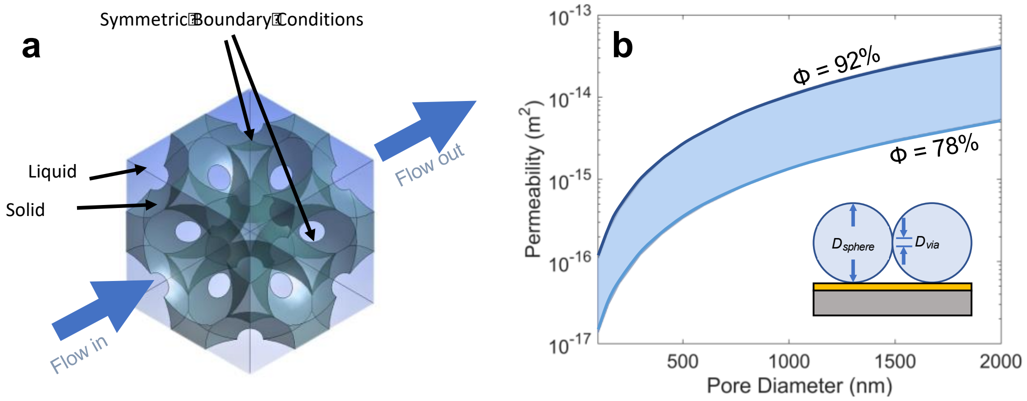

Figure S2. Unit cell showing geometry and boundary conditions for permeability calculations. (a) The fluid is introduced using 0.01 m/s velocity inlet (left face) and pressure outlet boundary conditions (right face). The other

surfaces are set to have symmetric boundary conditions. The gray is the solid domain and blue is the liquid domain. **(b)** Based on computational analysis, permeability increases with pore diameter and varies depending on the porosity. The porosity (78% to 92%) is calculated based on the degree of sphere overlapping (2% to 10%).

3. Statistics of Crystalline Porous Grain Width and Interconnecting Via

The capillary performance parameters of different pore diameters are shown to vary significantly within an order of magnitude. In order to elucidate further understanding of porous structural effects on wicking capabilities, we measure the crystalline grain width in Fig. S3a-c and the normalized via diameters of the copper IOs that are used in the wicking experiments in Fig. S3d-f. The large variance of capillary performance of the samples can be explained by the wide range of D_{via}/D_{pore} due to potential non-uniform annealing. For instance, the discrepancy for the K/R_{eff} of 1000 nm pore diameter samples can be attributed to the spread of normalized via diameters to their pore diameters, which reveals three different sets of porosity level between the three samples studied. The distribution of grain width also shows a gradual increase with pore diameters.

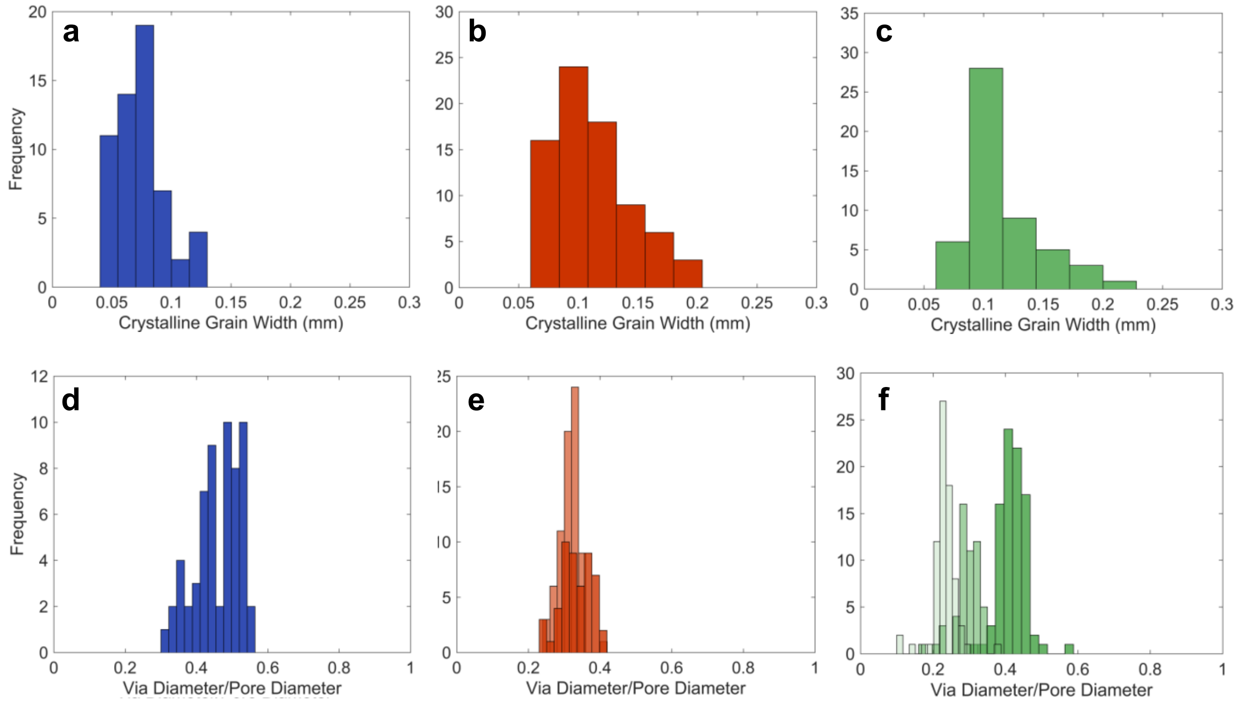

Figure S3. Statistical analysis of grain width and normalized via diameter for varying pore diameters. The frequency count of **(a-c)** crystalline grain widths and **(b)** normalized via diameters for 300, 600, and 1000 nm pore diameter samples as indicated by the blue, red, and green bar graph, respectively. Increasing in pore diameters result in slightly larger crystalline grain widths. The wide range of via diameters for 1000 nm pore diameter samples is attributed to the non-uniform annealing of the samples, which result in three different sets of via diameters that can be seen between three different samples (as indicated by the degree of color transparency).

4. Gradient Multilayered Copper Inverse Opal: Thickness and Capillary Performance

Gradient Copper IOs are used in capillary wicking experiment. In order to ensure that variation in layer thicknesses does not affect the capillary performance parameter, SEM is used to characterized the thickness of each layer of 300, 600, and 1000 nm pore diameter. The majorities of individual layer are within ~7-10 µm in thickness, and the thickness of individual layer is indicated with different colors in the bar graph.

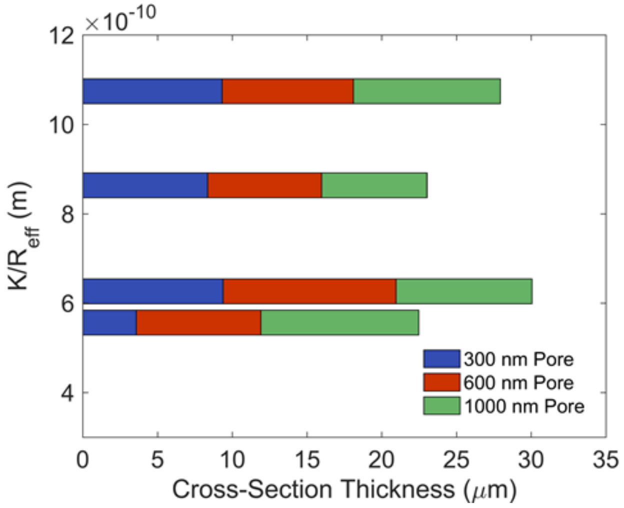

Figure S4. The thickness of and capillary performance of gradient copper inverse opals. The measured capillary performance of gradient-multilayered copper IOs. The stacking arrangement of these gradient IOs have pore diameters of 300, 600, and 1000 nm is examined using SEM to quantify the thickness of individual layers, which are within ~7-10 µm in thickness and are indicated as different colors in the bar graph. The K_{eff}/R_{eff} values of gradient IOs are in the range of ~0.6-1.1 x 10^{-3} µm.

5. Fabrication of Multilayered Copper Inverse Opals with Graded Pores

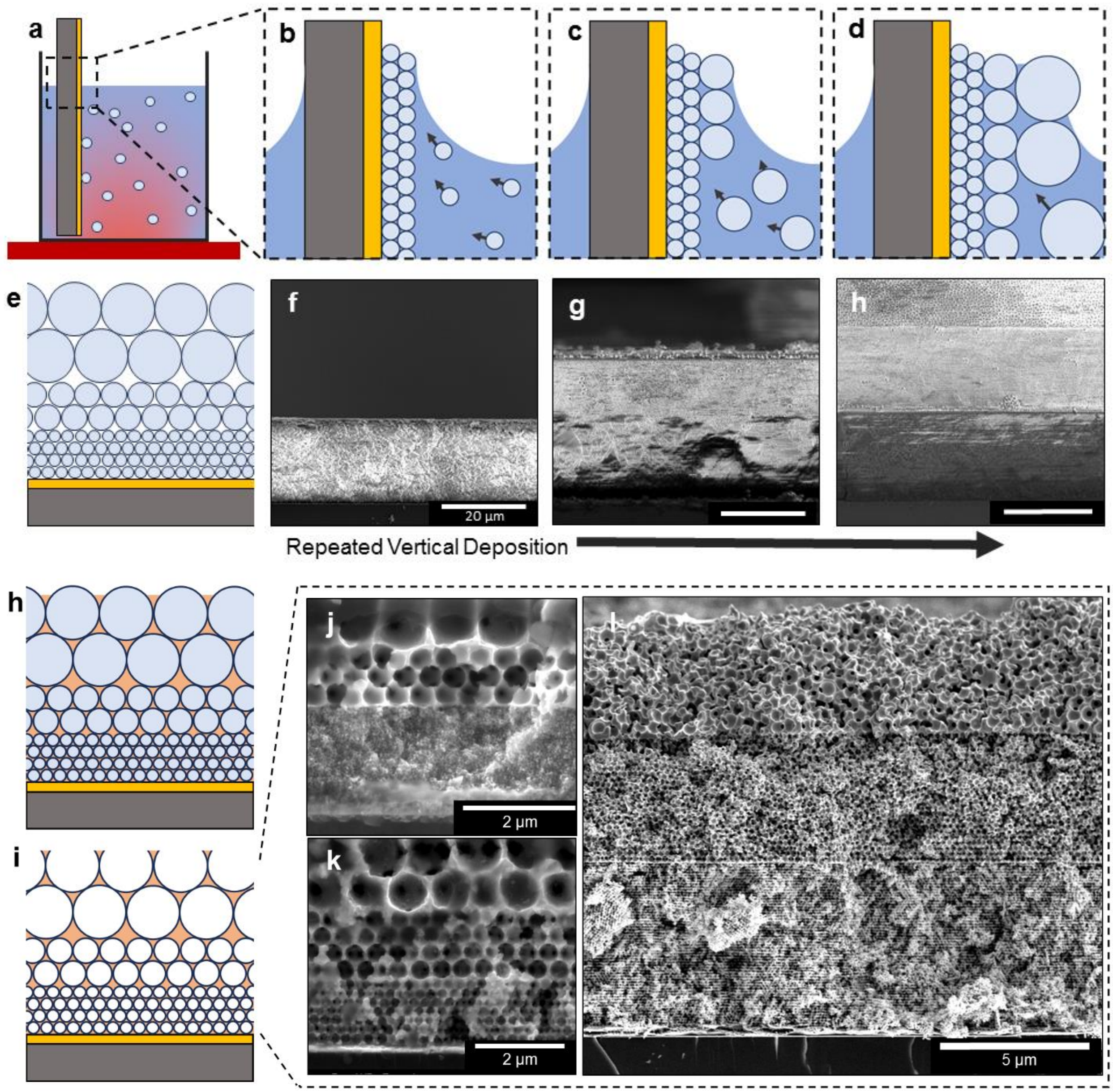

Figure S5. The fabrication process for creating monoporous and gradient copper inverse opals. (a) A substrate with gold patterns is vertically placed into a well of colloidal suspension that is heated at the base. As the suspension evaporates, thin film of opals deposits at the substrate-meniscus contact region. Monoporous IOs only require one deposition process while **(b-c)** gradient structure requires repeated trials in order to stack different sphere diameters. The resulting stacked spheres are shown as a diagram in **(d)** and SEM images in **(e-g)**. After **(h)** electroplated copper infiltrates the void volumes between the spheres, **(i)** the sacrificial spheres are removed to reveal IOs with various combination of gradient spatial arrangement, such as **(j)** 60, 400, and 1000 nm; **(k)** 200, 400, and 1000 nm; **(l)** 300, 600, and 1000 nm.

6. Opal Self-assembly via Van der Waals Forces.

The opal self-assembly process is sensitive to the process temperature, colloidal concentration, and sphere size on the number of deposited opal layers. Smaller colloids are shown to be more sensitive to concentration changes than larger spheres, as shown in Fig. S6a. This may be because at the same mass concentration, there are higher counts of smaller spheres present in comparison to larger, heavier spheres. The results also show that smaller spheres are less prone to sedimentation and that gentle convective mixing helps resupply the meniscus-substrate-contact region with a sufficient supply of colloidal particles for thicker deposition. The induced gentle convective mixing is controlled by the base heating of the colloidal suspension. Various process temperatures are analyzed to control evaporation rate and opal film thickness (Fig. S6b). Based on the results, an optimal process temperature of ~55°C is used for the remaining of the experiments in order to produce uniform opal film thickness. By considering the evaporation rate to be equal to the colloid film growth rate during vertical deposition, we can predict the thickness of as-produced multilayer opals according to[4,5]:

$$n = \frac{\beta\, L\, \varphi}{0.605\, d\, (1-\varphi)} \tag{S2}$$

where n is the number of layer, L is the meniscus height, β is the ratio between the velocity of a particle in solution and fluid velocity and is taken to be unity[4], d is the particle diameter, and φ is the particle volume fraction in the solution. The thickness data presented in Fig. S6a shows a good agreement with the model (dashed line). In addition, the equation also validates our theory that evaporation rate, as controlled by the base heat temperature, is not a major factor in determining opal deposition thickness.

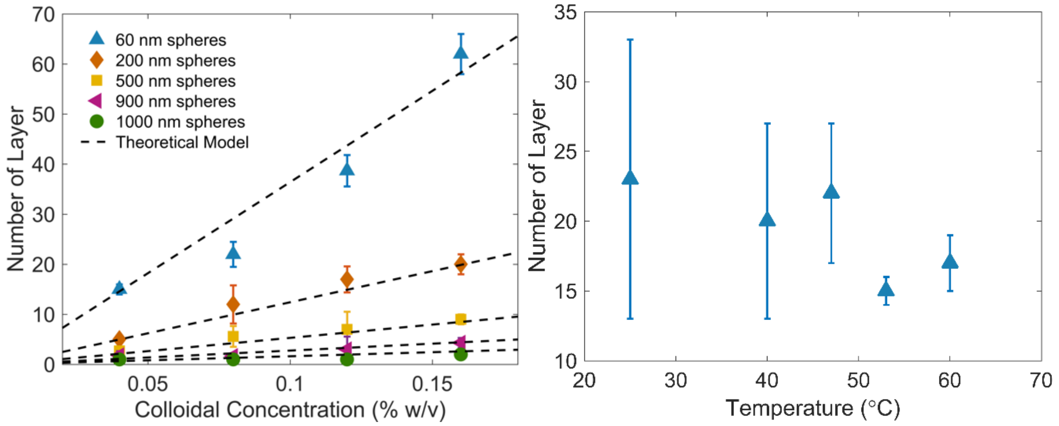

Figure S6. Controlling opal film thickness, quality, and consistency. (a) Variation of colloidal concentration on the number of deposited opal layers for different monodispersed sphere sizes with a base heating temperature of 55 °C. The dashed line indicates expected thickness of the opal film, based on equation (S2). It is essential to provide the heating to the colloidal suspension during vertical deposition to fabricate uniform self-assembly of sacrificial spheres. This heating induces convective mixing that prevents sedimentation of the colloidal particles, resulting in controlled opal deposition thickness. Hence, the optimum temperature of ~55°C is selected to yield the most consistent and crystalline opal formation. **(b)** The plot represents the opal deposition at various base heating temperature for 60 nm sphere diameter at 0.04 %w/v concentration.

References

[S1] Darcy, H. *Les Fontaines Publiques de la Ville de Dijon*. (Dalmont, 1865).

[S2] Xu, P. & Yu, B. Developing a new form of permeability and Kozeny–Carman constant for homogeneous porous media by means of fractal geometry. *Advances in Water Resources.* **31,** 74-81 (2008).

[S3] Bourbie, T., Coussy, O., Zinszner, B. and Junger, M.C. *Acoustics of Porous Media*. (CRC Press, 1992).

[S4] Dimitrov, A. S. & Nagayama, K. Continuous convective assembling of fine particles into two-dimensional arrays on solid surfaces. *Langmuir.* **12,** 1303-1311 (1996).

[S5] Jiang, P. *et al.* Single-crystal colloidal multilayers of controlled thickness. *Chem. Mater.* **11,** 2132-2140 (1999).
